# Effects of methane emissions on multiple myeloma-related mortality rates: A World Health Organization perspective

**DOI:** 10.1097/MD.0000000000037580

**Published:** 2024-04-12

**Authors:** Can Özlü, Cumali Yalçin

**Affiliations:** a Internal Diseases, Hematology, Medicine Faculty, Kutahya Health Sciences University, Kütahya, Turkey.

**Keywords:** methane emission, mortality, multiple myeloma

## Abstract

In this research, it was aimed to evaluate effects of methane emissions on multiple myeloma related mortality rates. Two countries in Europe (Germany and Netherlands) and 1 country for each region (Turkey, USA, Brazil, Egypt, and Australia) were selected within The World Health Organization Database. Multiple myeloma mortality rates of countries between 2009 and 2019 were used as dependent variable of the research. Methane emission level and agriculture methane levels of countries were used as independent variables from The World Bank Database. Current health expenditure and healthy life expectancy were used as controlling variables. Multiple myeloma-related mortality rate was the highest in the USA, followed by Germany, Brazil, Turkey, Australia, Netherlands, and Egypt. Difference analysis results were significant (*P* < .05). Methane and agriculture methane emissions were the highest in the USA. Multiple myeloma mortality was positively correlated with methane emissions (*R* = 0.504; *P* < .01), agricultural methane emissions (*R* = 0.705; *P* < .01), and current health expenditure (*R* = 0.528; *P* < .01). According to year and country controlled correlation analysis results, multiple myeloma mortality (MMM) was positively correlated with methane emissions (*R* = 0.889; *P* < .01), agricultural methane emissions (*R* = 0.495; *P* < .01), and current health expenditure (*R* = 0.704; *P* < .01). Methane emission (B = 0.01; *P* < .05), Germany (B = 9010.81; *P* < .01), the USA (B = 26516.77; *P* < .01), and Brazil (B = 4886.14; *P* < .01) had significant effect on MMM. Nonagricultural methane production has an increasing effect on MMM. Therefore, by looking at the differences between agricultural methane emissions and general methane emissions, studies can be conducted that allow for more effective global comparisons.

## 1. Introduction

Malignant plasma cells, which are typically linked to monoclonal protein in serum or urine, are the hallmark of multiple myeloma (MM). As the disease progresses, plasma cells can also be seen in peripheral blood or extramedullary regions.^[1]^ Roughly 10% of all hematologic malignancies and 1% of all cancers are caused by MM.^[2]^ Prior to the turn of the century, the majority of MM patients died just a few years following their diagnosis. However, over the previous 20 years, achievements have significantly improved.^[3]^ An established therapy objective for MM is to target the immune system; early methods included immune stimulants like interferon and allogeneic hematopoietic stem cell transplantation, along with the graft versus myeloma effects that went along with it.^[4]^ Therapies have been included in the best therapeutic method now available, which employs treatment cassettes with non-cross-reacting, synergistic medicines administered in phases for treatment.^[5]^

With 439 kJ mol energy required to dissolve the hydrocarbon bond, methane is the most abundant, totally reduced, and stable type of hydrocarbon. Although methane is a commonly used energy source, it contributes to global warming second only to carbon dioxide.^[6]^ The warming potential of methane is far greater than that of carbon dioxide. Industry emissions account for a significant portion of methane emissions, some of which are now easily identified and reduced.^[7]^ Although atmospheric evasion can be decreased by aerobic methane oxidation, little is known about how much of it occurs or how to manage it.^[8]^ In 2021, atmospheric methane concentrations hit a record-breaking 1900 parts per billion, necessitating action to address all methane sources.^[9]^ Natural chemical and biological processes, such as reactions with air hydroxyl and chlorine, as well as methane-eating bacteria found in soil and water, all contribute to the destruction of methane.^[10]^ Although studies on methane reduction and global emission reduction are carried out due to global warming, studies on its direct effects on health are quite limited.

An essential component of the contacts between plasma cells and the extracellular matrix and bone marrow stromal cells is very late antigen-4 (VLA-4), a transmembrane adhesion receptor present on normal plasma cells. The peptidomimetic ligand for VLA-4 is called LLP2A. For the purpose of labeling cuprum-64, LLP2A was conjugated to 1,4,8,11-tetraazacyclotetradecane-1-(methane phosphonic acid)-8-(methane carboxylic acid) (CB-TE1A1P) chelators.^[10]^ High-affinity ligands that target their receptors make up the peptides employed as positron emission tomography radiotracers. The selective peptidomimetic ligand LLP2A has a strong affinity for the activated version of VLA-4. Viral cell adhesion molecule, which is expressed in bone marrow stromal cells, interacts with aberrant VLA4 expression in MM cells to improve cell adhesion-mediated drug resistance. The chelators CB-TE1A1P, which are defined as 1,4,8,11-tetraazacyclotetradecane-1-[methane phosphonic acid]-8-[methane carboxylic acid], were conjugated with LLP2A.^[11]^ In another study, Sood et al reported that Tc-99m-hydroxy-methane-diphosphonate bone scan results in diffuse and high hepatic uptake, hypoxia from respiratory failure, and the development of hepatic necrosis in MM patients.^[12]^ Adenosine monophosphate-activated protein kinase activator metformin has been demonstrated to have synergistic effects with dexamethasone against MM and to decrease myeloma proliferation through the IGF-1R/PI3K/AKT/mTOR pathway.^[13]^ Although there are no direct studies linking methane-containing compounds to MM, there may be an association in the context of ligand and cell adhesion-mediated drug resistance. Therefore, in this study, it was aimed to evaluate effects of methane emissions on MM related mortality rates.

## 2. Methods

### 2.1. Research sample

In the WHO Mortality Database, there are 6 areas as:

− Europe
− Asia
− North America and the Caribbean
− Central and South America
− Africa
− Oceania

In order to demonstrate WHO mortality database, 2 countries in Europe (Germany and Netherlands) and one country for each region (Turkey, the USA [The United States of America], Brazil, Egypt, and Australia) were selected. In country selection criteria, The World Bank data repository was also mentioned as a restriction criterion. For example, Mexico was not selected, since it does not have methane levels in the World Bank Data Repository. All countries had data for 2009 and 2019 time period. Independent and dependent variables of the research were selected as below:

### 2.2. Independent variable

MMM: Multiple Myeloma Mortality − WHO Mortality Database

### 2.3. Independent variables

METEM: Methane emissions (kt of CO_2_ equivalent) − The World Bank Database

AMETEM: Agricultural methane emissions (thousand metric tons of CO_2_ equivalent) − The World Bank Database

CHEXP: Current health expenditure per capita (current US$) − The World Bank Database

LEXP: Life expectancy at birth, total (years) − The World Bank Database

### 2.4. Statistical methods and rationale

The research data were described using lowest and maximum values, along with mean and standard deviation values. The groups’ variations in HGB over time were examined using parity analysis. Nonparametric tests were run since there were fewer than 30 statistical units in the time series. The relational screening analysis included a Spearman rho correlation analysis. The effect analysis involved the application of Generalized Linear Model (Logit) analysis due to linearization discrepancies.^[14,15]^ In the statistical analysis of the research parameters, normality distribution, standard analysis assumptions and statistical method approaches were determined for rationale. Cofounders are considered constant with respect to other variables because there are multiple countries and centers. In this respect, the research is limited to adiabatic. The SPSS 25.0 for Windows program was used for all analyses, with a 95% confidence interval and a significance level of 0.05.

## 3. Results

For difference analyses, the Kolmogorov Smirnov test was performed beforehand as a normality test and the Kruskal Wallis test was performed as a result. Multiple myeloma related mortality rate was the highest in the USA, followed by Germany, Brazil, Turkey, Australia, Netherlands, and Egypt. Difference analysis results were significant (*P* < .05). Methane and Agriculture Methane emissions were the highest in the USA (Table 1).

**Table 1:** Multiple myeloma related mortality rates and methane emissions of countries from 2009 to 2019.

Median (range)	MMM	METEM	AMETEM
Australia	2514.91 ± 194.31 2550.00 (2214.00–2845.00)	150,916.91 ± 15,892.34 151,225.32 (129,412.31–177,596.53)	87,160.14 ± 17,458.28 85,131.19 (68,008.25–118,990.14)
Brazil	7669.91 ± 875.03 7691.00 (6373.00–9135.00)	432,574.11 ± 10,413.20 433,815.23 (407,797.89–443,522.16)	333,462.15 ± 5012.37 334,263.76 (320,998.09–339,754.23)
Egypt	219.91 ± 85.08 193.00 (130.00–383.00)	64,973.51 ± 1570.50 65,216.61 (62,141.48–67,405.80)	14,943.68 ± 1929.60 15,570.99 (9870.73-16,730.34)
Germany	10,907.18 ± 572.63 10,876.00 (9753.00–11,643.00)	53,619.25 ± 3324.56 53,386.29 (48,449.87–58,902.25)	33,795.44 ± 838.62 33,981.64 (31,887.32–34,619.49)
Netherlands	2057.82 ± 157.34 2048.00 (1851.00–2290.00)	17,011.13 ± 831.33 17,181.46 (15,529.26–18,043.59)	12269.48 ± 504.03 12,165.16 (11,574.67–13,152.48)
Turkey	3032.00 ± 330.87 3238.00 (2483.00–3328.00)	44,990.30 ± 2310.57 44,562.39 (42,380.29–49,343.10)	19,336.13 ± 3097.50 20,101.75 (15,074.57–24,546.54)
The USA	33,820.00 ± 541.19 34,085.00 (32,803.00–34,409.00)	691,154.69 ± 42,709.52 683,241.85 (634,800.94–784,496.75)	196,766.19 ± 4200.54 198,063.90 (190,889.07–202,312.20)
*P* value*	<.05	<.05	<.05

AMETEM **=** agricultural methane emissions (thousand metric tons of CO_2_ equivalent), METEM **= **methane emissions (kt of CO_2_ equivalent), MMM = multiple myeloma mortality (person).

* Kruskal–Wallis test.

**Figure 1.**
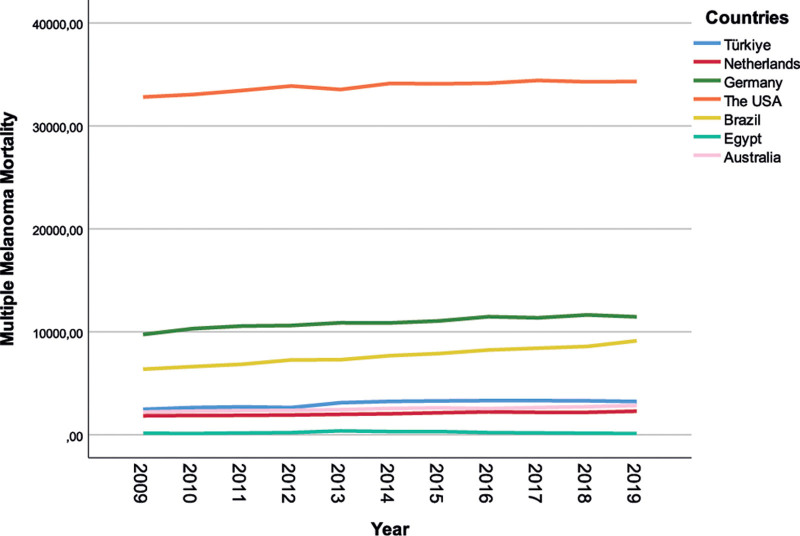
Multiple melanoma mortality according to year and countries.

The USA had the highest MMM rate, and all countries had slightly increasing trend within the time period of the research (Fig. 1).

Spearman rho correlation analysis results showed that multiple myeloma mortality (MMM) was positively correlated with methane emissions (*R* = 0.504; *P* < .01), agricultural methane emissions (*R* = 0.705; *P* < .01) and current health expenditure (*R* = 0.528; *P* < .01). Year and country controlled correlation analysis results showed that MMM was positively correlated with methane emissions (*R* = 0.889; *P* < .01), agricultural methane emissions (*R* = 0.495; *P* < .01), and current health expenditure (*R* = 0.704; *P* < .01) (Table 2).

**Table 2:** Spearman rho and year and country controlled partial correlation analysis results for multiple myeloma mortality and research parameters.

	Spearman rho		Year and country controlled	
	*P*	r	*P*	r
METEM	.504*	0.000	.889*	0.000
AMETEM	.705*	0.000	.495*	0.000
CHEXP	.528*	0.000	.704*	0.000
LEXP	.123	0.287	.160	0.170

AMETEM **=** agricultural methane emissions (thousand metric tons of CO_2_ equivalent), CHEXP **=** current health expenditure per capita (current US$), LEXP **=** life expectancy at birth, total (years), METEM **=** methane emissions (kt of CO2 equivalent), MMM **=** multiple myeloma mortality (person).

* *P* < .01.

Generalized Linear Model (Scale Logit) results showed that methane emission (B = 0.01; *P* < .05), Germany (B = 9010.81; *P* < .01), the USA (B = 26516.77; *P* < .01), and Brazil (B = 4886.14; *P* < .01) had significant effect on MMM (Table 3).

**Table 3:** Generalized linear model (scale logit) for effects of significantly correlated parameters on multiple myeloma mortality.

Parameter	*B*	Std. error	95% Wald confidence interval		Hypothesis test		
			Lower	Upper	Wald Chi-square	df	*P*
(Intercept)	923.95	747.17	−540.48	2388.37	1.53	1	.216
METEM	0.01	0.01	0.01	0.02	4.40	1	.036
AMETEM	−0.01	0.01	−0.02	0.01	0.87	1	.350
CHEXP	0.14	0.15	−0.16	0.44	0.86	1	.353
[Countries = Turkey]	1753.12	685.79	408.99	3097.24	6.53	1	.011
[Countries = Netherlands]	321.71	652.68	-957.51	1600.94	0.24	1	.622
[Countries = Germany]	9010.81	461.90	8105.51	9916.12	380.57	1	.000
[Countries = The USA]	26,516.77	1900.26	22,792.32	30,241.22	194.72	1	.000
[Countries = Brazil]	4886.14	2211.45	551.77	9220.51	4.88	1	.027
[Countries = Egypt]	−1228.21	785.91	−2768.56	312.14	2.44	1	.118
[Countries = Australia]	0						
(Scale)	163,728.62	26,387.26	119,382.607	224,547.47			

AMETEM = agricultural methane emissions (thousand metric tons of CO_2_ equivalent), CHEXP = current health expenditure per capita (current US$), METEM = methane emissions (kt of CO_2_ equivalent), MMM = multiple myeloma mortality (person).

## 4. Discussion

Multiple myeloma is a type of cancer consisting of white blood cells called plasma cells,^[16–18]^ and plasma cells increase uncontrollably, as in other types of cancer.^[19–23]^ Although there have been serious advances in the field of health and especially in the cancer diagnosis and treatment process, deaths due to MM are still considered an important public health problem.^[19–23]^ Although there are many risk factors in deaths caused by MM, the number of studies focusing on environmental factors is quite limited. Methane emission is a subject studied in areas such as global warming,^[24,25]^ especially the marine environment and ocean fauna.^[24,26]^ On the other hand, there are no sufficient studies on the effect of methane emission levels, especially MMM, on health.

Although there have not been direct relationship between methane emissions and MM, there are some ligand and cell related transition stages which may indicate indirect effects of methane on MM. The activated form of VLA-4 binds well to the selective peptidomimetic ligand LLP2A. To enhance cell adhesion-mediated drug resistance, abnormal VLA4 expression in MM cells interacts with viral cell adhesion molecule, which is produced in bone marrow stromal cells. LLP2A^[11–13]^ was conjugated with the chelators CB-TE1A1P, which are specified as 1,4,8,11-tetraazacyclotetradecane-1-[methane phosphonic acid]-8-[methane carboxylic acid].

Although there is no direct relationship between MMM and methane emission in the literature, there are studies indicating MMM and methane emission on a ligand and chelate basis.^[10,13]^ In these studies, methane emission is included in the chemical process as a binder or ligand. However, in a study conducted in a different field but similar in method, Torun et al^[25]^ reported that nitrite emissions were associated with deaths caused by tuberculosis. According to our results, the USA is the region where the most methane gas is produced and the most deaths due to MM occur. Germany follows this. Egypt is the country with the least methane emissions and the least MM deaths. In fact, the first question here is to what extent MM-related deaths are diagnosed in countries such as Egypt and to what extent methane emissions can be monitored. However, the research was conducted for 6 different countries, the data set is one of the most respected data sets in the world, and there is possible data pollution for both variables. In other words, a lack of data or disruptions in information sharing in deaths caused by MM may also cause disruptions in methane emission data. Therefore, it is possible to state that the deviation margins of the research results are similar.

According to the results of the correlation analysis, both general methane emission level and agricultural methane emission levels had a statistically significant relationship with MMM in correlations without year and country control. Although per capita health expenditures were also significantly related to MMM, the relationship between healthy life expectancy from birth and MMM was not significant. In the year and country controlled correlation, while the methane emission effect was increasing, the effect of agricultural methane emission was decreasing. The impact of health expenditures also increased in the controlled regression. This indicates that methane emissions from industry are more effective, and in countries such as the USA and Germany, this negative effect is covered by health expenditures. Although there are no studies in the literature directly revealing the relationship between methane and MM, there are studies involving methane compounds as a mechanism.^[11–13]^ This situation also supports the research findings. However, further research is needed for the mechanism of correlation analysis results. In this respect, our study can be considered a pioneering study.

According to multivariate analysis results, the effect of methane emissions on MMM was significant for the USA, Brazil and Germany, but this effect was not valid for agricultural methane. This indicates that methane emission values in developed countries may be the cause of MMM. At this point, it can be argued that the incidence of MMM is high in Germany due to the elderly population. However, the same situation is not the case for the USA; there is a structure that includes all age groups. In addition, the congenital healthy life expectancy parameter was included in the analysis as a control variable to prevent the negative effects of factors such as country age, demographic variables, and health system changes from distorting the analysis. It is therefore possible to argue that these results are relatively free from the distorting effect of demographic differences.

### 4.1. Limitations of the study

Although MM has been the subject of many studies from past to present, these studies are generally within their own field and the number of multidisciplinary studies is quite low. For this reason, it may be possible not to evaluate the effect of a chemical process, mechanism or compound that may actually explain a mechanism related to MM. The biggest limitation in the research is that the studies on methane mostly focused on environmental pollution and global warming. However, considering the effects of chelates and ligands on health, there are not enough studies.

Another important limitation of the research is that data on methane emissions cannot go back very far, since environmental issues are also related to industrial production and economic reasons. Even today, in some countries, especially Turkey, there is no data after 2019. Therefore, there is a limitation of data in terms of vertical series analysis in the research. In order to eliminate this gap, a data set unit of 60 series, 6 countries and 10 years, was created.

Although research findings have revealed the relationship between methane emissions and MM, generalizing this situation may cause some bias. The fact that not every country has methane emission data, inadequate methane emission measurements and errors in this regard may cause deviations from the research results. However, existing data indicate that this relationship may be strong.

### 4.2. Contributions of the research to the literature

The most important contribution of the research to the literature is that it is multidisciplinary and therefore, by addressing environmental issues and health issues in different fields under public health, it has opened a new horizon for further research in both different fields and field applications in public health. Looking at environmental issues from only one perspective, as in this example, looking only at global warming, will cause the other effects of the issue to not be seen in a global sense. In this respect, the research can make significant contributions to the field in a holistic, multidisciplinary sense.

Another contribution of the research to the field is that it shows and reveals from a world perspective how important environmental factors are among the risk factors for MM. In this context, research is important as it reveals the necessity of a global fight against MM and similar diseases.

Based on these findings, public and academic studies, especially on environmental gas emissions, should be encouraged and advanced data sharing and analyzes should be developed on this subject. In this respect, the research will guide the studies that can be done in this field.

## 5. Conclusion

According to the results obtained in the study, there is a statistically significant and strong relationship between industrial and general methane emissions and MMM, rather than agricultural methane emissions. Since the research results include samples representing all world regions on the WHO list, it is possible to evaluate the results globally and argue that nonagricultural methane production has an increasing effect on MMM. Therefore, by looking at the differences between agricultural methane emissions and general methane emissions, studies can be conducted that allow for more effective global comparisons. Additionally, the results of these studies can be examined in different disciplines in the field of health.

## Acknowledgments

We thank Kadir Yilmaz with his valuable statistics support.

## Author contributions

**Conceptualization:** Can Özlü.

**Data curation:** Can Özlü.

**Formal analysis:** Can Özlü.

**Writing – original draft:** Can Özlü, Cumali Yalçin.

**Writing – review & editing:** Cumali Yalçin.
